# Chemical composition and antifungal activities of aromatic water of *Zataria multiflora* Boiss.

**DOI:** 10.18502/CMM.7.3.7255

**Published:** 2021-09

**Authors:** Ali Arabi Monfared, Maryam Yazdanpanah, Zahra Zareshahrabadi, Keyvan Pakshir, Mehdi Ghahartars, Davood Mehrabani, Samira Yazdanpanah, Aida Iraji, Kamiar Zomorodian

**Affiliations:** 1 Central Research Laboratory, Shiraz University of Medical Sciences, Shiraz, Iran; 2 Department of Medical Mycology and Parasitology, School of Medicine, Shiraz University of Medical Sciences, Shiraz, Iran; 3 Basic Sciences in Infectious Diseases Research Center, School of Medicine, Shiraz University of Medical Sciences, Shiraz, Iran; 4 Molecular Dermatology Research Center, School of Medicine, Shiraz University of Medical Sciences, Shiraz, Iran; 5 Stem Cell Technology Research Center, School of Medicine, Shiraz University of Medical Sciences, Shiraz, Iran; 6 Stem Cells Technology Research Center, Shiraz University of Medical Sciences, Shiraz, Iran

**Keywords:** Aromatic water, Antifungal property, *Candida albicans*, Essential oil, *Zataria multiflora* Boiss

## Abstract

**Background and Purpose:**

In Iranian culture, aromatic waters harboring a slight amount of essential oil have been popularly used for many years as a pleasant non-alcoholic drink with various medicinal properties.

In this study, chemical composition of *Zataria multiflora* Boiss. (ZM) aromatic water was determined and its *in vitro* and in vivo antifungal properties were investigated.

**Materials and Methods:**

Chemical composition of the essential oil extracted from aromatic water (AW) of ZM was analyzed by Gas chromatography and gas chromatography-mass spectrometry (GC-MS). The antimicrobial activity of the AW against *Candida* species was determined by broth micro-dilution methods. Additionally, biofilm formation inhibition and antioxidant activity of the AW were measured using XTT reduction and DPPH methods, respectively. Antifungal activities of the AW in the prevention and/or treatment of gastrointestinal (GI) candidiasis in animal models were also evaluated.

**Results:**

The GC-MS analysis revealed that the major constituents of ZM AW were Carvacrol (46.56%) and Thymol (40.67%). The ZM AW inhibited the growth and biofilm formation of *Candida* species in the range of 0.25-0.5 V/V. Moreover, ZM AW significantly decreased *Candida* colonization in therapeutic groups (*P*<0.05).

**Conclusion:**

Given the wide therapeutic potential of ZM AW, including antifungal and antioxidant activities, it might be possible to use it in the management of mucocutaneous or alimentary candidiasis.

## Introduction

Aromatic plants have been utilized in health and aromatherapy for thousands of years, as well as in the cosmetics and food sectors [ 1 ]. Many of these plants and their aromatic compounds have already been demonstrated to have therapeutic effects [ 2 - 4 ]. Aromatic waters (AWs) harboring a slight amount of essential oil (EO) are therapeutic distillates of aromatic plants. In folk medicine, the AWs are popularly used as a pleasant non-alcoholic drink for their medicinal properties. The effective component of AWs is EO, which contains monoterpenes with different medicinal properties [ 1 ].

The *Lamiaceae* are a large family with about 4,000 species divided into 200 genera. Many species of the *Lamiaceae* family are used in the medicine, culinary, and cosmetic industries. The *Zataria multiflora* (Boiss ZM) is a thyme-like EO-bearing plant that grows as an endemic, perennial, and bushy fragrant herb in the central and southern regions of Iran, Pakistan, and Afghanistan. [ 5 ]. This plant is often used as a flavoring and fragrance ingredient in a wide range of foods and beverages [ 6 ]. Several studies have shown that ZM EOs and extracts can stimulate innate immunity [ 7 ] and exhibit antibacterial and anti-inflammatory properties [ 8 - 10 ]. In previous studies, the EOs distilled from ZM exhibited considerable antifungal activities [ 11 , 12 ]. Furthermore, ZM EOs have been found to suppress radial fungal growth and aflatoxin generation by *Aspergillus flavu*s in cheese [ 6 ]. Nowadays, the incidence of resistance to routine antifungal drugs, especially against *Candida* species, is challenging in the management of candidiasis. To address this issue, it is suggested to use new antifungal drugs, particularly those derived from natural sources [ 13 ].

There is little known about the composition and antibacterial properties of ZM aromatic water, a common non-alcoholic herbal drink. As a result, the current research was designed to assess the chemical composition, antioxidant, antifungal, and anti-biofilm properties of ZM AW against *Candida* species. Furthermore, the therapeutic effects of this AW in the treatment of GI candidiasis were investigated.

## Materials and Methods

In this research, we applied the same techniques as Saharkhiz et al. [ 4 ] 

### Plant material and essential oil extraction from *Zataria multiflora* Boiss aromatic water

The AW used in this study was provided by the Nab Factory (Meymand, Iran). An experienced botanist identified and verified the ZM used for AW extraction. The voucher specimen was stored in the herbarium (Voucher no. HSUMS 301). For this reason, AW which is also called herbal water was decanted three times with 300 ml of diethyl ether as an extracting solvent using a separatory funnel. During this process, the EO of the AW was transferred from the aqueous phase to the diethyl ether phase. Diethyl ether phase was dried by anhydrous sodium sulfate and the solvent was evaporated at room temperature to produce concentrated EO. It was stored at 4 °C in dark until further analysis.

### Identification of the *Zataria multiflora* Boiss aromatic water essential oil compounds

The EOs were analyzed by an Agilent Technologies 7890A gas chromatograph series coupled with a 7000 Triple Quad mass spectrometer. The operating mode was EI at 70 eV. The flow rate of helium, as the carrier gas, was maintained at 1.2 ml/min. The injector and auxiliary temperatures were kept at 250 °C and 280 °C, respectively. Separation was performed using a DB-1MS capillary column (30 m, inside diameter: 0.25 mm; film thickness: 0.25 µm).

The oven temperature was set to rise from 60 to 280 °C at a rate of 4 °C/min and then stay there for 4 min. Moreover, 0.1 µl of EO was injected in a split mode with a split ratio of 1:30 [ 14 ], and the mass spectra were taken between 46 and 650 amu.

Identification of the constituents of EOs was based on the comparison of the mass spectra and retention indexes with those reported in the Wiley Library, the NIST mass spectral library, and Adams (2001). The relative percentage peak areas of the individual compounds were obtained for comparing the samples.

The linear retention indices for all compounds were measured using the Van Den Dool method, which involved injecting a solution containing the homologous sequence of C8-C26 n-hydrocarbons after injecting EOs under the same chromatographic conditions. The device software determined relative percentage amounts from the total area under the peaks.

### Antioxidant assays

The DPPH (2,2-diphenyl-1-picryl-hydrazyl-hydrate) radical scavenging activity was measured using the previously published methods. In total, 270l of various EO dilutions were combined with a 100 M methanolic solution of DPPH and incubated at room temperature for 30 min. When the odd electron of the nitrogen atom in DPPH interacts with an antioxidant molecule capable of donating either hydrogen or electron, it is reduced to the corresponding hydrazine derivatives. An ultraviolet-visible spectrophotometer was used to measure the color changes (from deep violet to bright yellow) at 517 nm. Curve-Expert (for Windows, version 1.34) was used to compute antioxidant levels and the percentage of inhibition for the AWs.

### Evaluation of the *in vitro* antifungal activities

#### Microorganisms

The antifungal activities of the AW were determined against 16 standard strains of *Candida*, including *C. ablicans* (CBS 5982, CBS 1912, CBS 562, ATCC 10261, CBS 2730), *C. tropicalis* (ATCC 750), *C. krusei* (ATCC 6258), *C. glabrata* (CBS 2192, CBS 2175, CBS 6144, ATCC 90030), *C. dubliniensis* (CBS 8501, CBS 8500, CBS 7987, CBS 7988), and *C. parapsilosis* (ATCC 4344). The antifungal susceptibility of the tested fungi against fluconazole was determined by the microdilution method [ 15 , 16 ]. 

### Determination of minimum inhibitory concentration

The minimum inhibitory concentrations (MIC) of AW against *Candida* species were determined using the broth micro-dilution method according to the clinical and laboratory standards institute. The RPMI-1640 (without bicarbonate and with l-glutamine) (Sigma, USA) was prepared and buffered at pH 7.0 with 0.165 mol of 3-(N-morpholino) propane sulfonic acid (MOPS) (Sigma-Aldrich, Steinheim, Germany). In 96-well microtitre trays, serial dilutions of the AW (1/2 to 1/1024v/v) were prepared using RPMI-1640 (Sigma, St. Louis, USA) buffered with MOPS (Sigma, St. Louis, USA). Fluconazole twofold dilutions were also prepared for the tested fungi, with final concentrations ranging from 0.25 to 128 g/ml.

The yeast inoculates were prepared from 24-h Sabouraud dextrose agar (SDA) (Merck Co., Germany) cultures by suspending the colonies in sterile normal saline and adjusting the turbidity of the inoculums to 0.5 McFarland standards at 530 nm wavelengths (this yields a stock suspension of 1-5 106 cells/ml). The working suspension was prepared by diluting the stock suspension by 1/1000 with RPMI. The trays were incubated at 30 °C for 24-48 h after 0.1 ml of the inoculums was added to the wells. As a sterility control, 200 µl of uninoculated medium were included (blank). Furthermore, growth controls (medium with inoculums but no AW or fluconazole) were included. The growth of each well was compared to the growth of the control well. The MIC was visually determined and defined as the lowest concentration of the AW that produced no visible growth. It should also be mentioned that each experiment was carried out three times. Furthermore, the minimum fungicidal concentrations (MFCs) of the tested AWs were determined by culturing 10 µL from the wells with no visible growth onto SDA plates. The MFCs were found to be the lowest concentration, yielding no more than four colonies and resulting in the mortality of 98% of the fungi in the initial inoculums [ 17 ].

### Inhibition of biofilm formation

According to a study performed by Ramage et al. [ 18 ], *Candida albicans* (ATCC 10261) were maintained on SDA plates. After 48 h, one colony loop was transferred to 20 ml Sabouraud dextrose broth (Merck Co., Germany) in a falcon tube and incubated overnight in an orbital shaker (100 rpm) at 30 °C under aerobic conditions. Yeast cells were harvested and washed twice in sterile phosphate-buffered saline (0.8%. Moreover, the cell densities were adjusted to 1106 cells/mL. The AWs were serially diluted in RPMI 1640 (1/2-1/1024v/v) on sterilized, polystyrene, flat-bottom 96-well microtiter plates (Nunc) [W/V], sodium chloride [Merck]; 0.02% [W/V], KH_2_PO_4_ [Merck]; 0.31% [W/V], Na_2_HPO_4_+12H_2_O [Merck]; 0.02% [W/V], potassium chloride [Panreac]; pH 7.4). After counting with a hematocytometer, the cells were resuspended in RPMI 1640 supplemented with L-glutamine (Gibco) and buffered with MOPS. The trays were incubated at 30 °C for 24-48 h in a humid environment after 0.1 mL of yeast inoculums were added to the wells. As a negative control, 200 µL of uninoculated media was added (blank). Additionally, RPMI containing yeasts but no AWs was used as a positive control.

A 2, 3-bis (2–methoxy–4–nitro–5–sulfo-phenyl)-2H-tetrazolium-5-carboxanilide (XTT) reduction test was used to provide a semi-quantitative assessment of biofilm development. The XTT (Sigma Chemical Co.) was prepared as a saturated solution in Ringer's lactate at a concentration of 0.5 mg/ml. This solution was sterilized with a 0.22-m-pore-size filter, split into aliquots, and kept at -70 °C. An aliquot of the XTT stock solution was frozen and treated with menadione sodium bisulfate (Sigma Chemical Co.) to achieve a final concentration of 1 µM of menadione before each test. After that, a 100 µL aliquot of XTT-menadione was added to each pre-washed well. The plates were incubated in the dark for 2 h at 37 °C before measuring the colorimetric change at 490 nm with a microplate reader (POLARstar, Omega, BMG LABTECH, Germany) [ 18 , 19 ].

### Therapeutic trials

The study was conducted on 27 female 4-week-old BALB/c mice (approximate weight 19-23g) who were maintained at 25-30 °C and fed a standard diet. To examine the effect of ZM AW on alimentary candidiasis, the mice were divided into three groups, one of which was the test group (9 mice) that received ZM AW (0.28 mg/mL). The other two groups were the positive control groups (9 mice) receiving 1 g/ml fluconazole (Sigma, USA) in their drinking water, and the third group (negative control, 9 mice) received just drinking water. As the healthy mice are resistant to yeast colonization (C. albicans), antibiotics and immunosuppressive medications were given prior to inoculation. The mice were given antibiotics (tetracycline [Sigma, USA] 1 g/liter, gentamicin [Sigma, USA] 0.1 g/liter, and streptomycin [Sigma, USA] 2 g/liter) three days before the yeast cell suspension was inseminated.

Afterward, the feeding needle was used to inject 100 ml of the yeast cell suspension with a concentration of 2×10^8^ cells into the stomach of each mouse. The animals were administered with cyclophosphamide (Sigma, USA) (100 mg/kg) immediately after the yeast gavages. The injections were administered again one week before the sacrifice [ 20 ]. At the end of the third, fourth, fifth, and sixth weeks following the fungal infection, the mice (three animals from each group) were killed by ketamine (Sigma, USA) overdose. 

The stomach was removed, and a portion of the stomach specimen was formalin-fixed for histological investigations, while another portion was weighed under sterile circumstances and homogenized in a tube containing sterile phosphate-buffered saline (500 mg/ml) with a homogenizer (Speed Mill plus-Analytik Jena, Germany). The homogenized specimens were cultured immediately on SDA plates. The colony forming units (CFUs)/500 mg were measured and compared after 24-48 h of incubation at 32 °C. The colonies in each group were counted and compared. All procedures were carried out in compliance with the rules of the Iranian Ministry of Health and Medical Education for animal care and management, as well as international conventions on animal experimentation [ 21 ].

### Histopathological evaluation

After the animals were euthanized and sacrificed, the tissue specimens from the digestive system, including the stomach and intestine, were placed into 10% buffered formalin for further fixation. The materials were processed routinely, and a 5 µm thick tissue section was produced and stained. For staining the specimens, hematoxylin and eosin (H&E), periodic acid–Schiff, and Gomori methenamine silver were employed, and the samples were seen under a light microscope (Olympus BX63). The presence or absence of fungal components, as well as any inflammatory reaction, was assessed [ 22 ].

### Statistical analysis

The independent t-test was used for the statistical analysis of differences among the groups. To compare the findings of the investigated groups based on sample time, the Mann-Whitney test was employed. A p-value of less than 0.05 was considered statistically significant. All statistical analyses were performed in SPSS software (version 18.0).

## Results

### Chemical compositions and antioxidant activity

The extracted EO of ZM was fragrant and yellow, but the AW was colorless with less fragrance. The EO was obtained at a yield of 0.28 mg/mL. The qualitative and quantitative evaluations of the EO extracted from AW of ZM are summarized in Table 1 where the compounds are listed according to their elution order.

**Table 1:** Chemical composition of essential oil extracted from aromatic water of *Zataria multiflora* Boiss.

Sample no.	Constituent	Calculated-RI	Area (%)
1	Cyclohexanone (3-methyl)	924.68	0.06
2	Octanol (3-)	982.76	0.03
3	Cineole1,8	1024.441	0.31
4	Fenchone	1070.559	0.49
5	Linalool	1085.941	1.33
6	Borneol	1150.289	0.23
7	Terpinen-4ol	1163.079	0.46
8	Terpineol-α	1173.526	0.79
9	Carvone	1218.341	4.44
10	Anethole-E	1260.341	2.5
11	Thymol	1271.976	40.67
12	Carvacrol	1281.878	46.56
13	Piperitenone	1307.61	0.97
14	Thymol acetate	1325.6	0.09
15	Piperitenone oxide	1329.425	0.19
16	Italicene ether(10-epi)	1492.237	0.7
17	Spathulenol	1557.947	0.12
18	Dill apiole	1586.895	0.06

RI: Retention index literature comparison. %: Relative percentage of *Zataria multiflora* AW Boiss essential oil constituents

The EO included a total of 18 compounds, accounting for 99.8% of the total volatiles. According to the gas chromatography-mass spectrometry (GC-MS) analysis, the major components of the EO were carvacrol (46.56%) and thymol (40.67%). The antioxidant activity of the EO was determined using the DPPH test. The DPPH is neutralized in this method by obtaining an electron or a hydrogen atom from antioxidant chemicals. The AW of ZM demonstrated modest antioxidant activity, neutralizing DPPH by up to 23.5% (23.450.76) at a dosage of 200 mg. Quercetin was used as a control antioxidant, and its IC50 value was 9.1±0.42µM.

### In vitro antifungal activities and inhibition of biofilm formation

Table 2 tabulates the antifungal activity of AW against the standard and clinical *Candida* strains. At a concentration of 0.25-0.50 V/V, AW of ZM inhibited the growth of the examined yeasts, including azole-resistant strains. Furthermore, the MFC of ZM AW ranged from 0.25 to 0.5 V/V. The XTT technique was used to assess the inhibitory effects of ZM AW on the development of biofilm by *C. albicans*. According to the results, *C. albicans* biofilm formation was reduced up to 70% at 0.5 V/V concentration.

**Table 2:** Antifungal effect of *Zataria multiflora* Boiss. aromatic water against *Candida* species.

Candida spp.	*Zataria multiflora*		Fluconazole
	MIC (v/v)	MFC (v/v)	MIC (µg/mL)
*C. albicans*	ATCC10261	0.25	0.5	16
*C. albicans*	CBS 562	0.5	0.5	0.25
*C. albicans*	CBS 5982	0.5	0.5	0.25
*C. albicans*	CBS 2730	0.5	0.5	0.5
*C. albicans*	CBS 1912	0.5	0.5	1.0
*C. dubliniensis*	CBS7987	0.5	0.5	1.0
*C. dubliniensis*	CBS 7988	0.5	0.5	1.0
*C. dubliniensis*	CBS 8501	0.5	0.5	1.0
*C. dubliniensis*	CBS 8500	0.5	G	0.25
*C. glabrata*	CBS 2175	0.25	0.5	0.25
*C. glabrata*	ATCC90030	0.25	0.5	0.5
*C. glabrata*	CBS 2192	0.25	0.5	0.25
*C. glabrata*	CBS 6144	0.25	0.5	0.5
*C. krusei*	ATCC6258	0.5	G	64
*C. parapsilosis*	ATCC4344	0.25	0.25	0.25
*C. tropicalis*	ATCC750	0.25	G	2

MIC: minimum inhibitory concentration, MFC: minimum fungicidal concentration, G: growth, ATCC: American Type Culture Collection, CBS: Centraalbureau voor Schimmelcultures

### Therapeutic trials and histopathological evaluation

After the third week of treatment, the CFUs obtained from cultured samples of the control (untreated) and AW groups were 400±70.7 and 300±100, respectively. As predicted, no fungal growth was detected in the culture of the fluconazole-treated mice. The independent sample t-test revealed a statistically significant difference (p-value= 0.02) between the control (untreated) and AW groups. On the fifth and sixth weeks, there was a significant difference between the control and AW groups (Table 3). The CFUs were considerably lower in the AW group compared to the control group, according to these findings.

**Table 3:** The colony forming units of the mice treated with aromatic water and controls based on sampling time.

Time		Control (untreated)	Aromatic Water	p-value	
Mean±SD	Mean±SD
Week3	400±70.70	300±10	0.285
Week4	265±21.21	115.21±17.69	0.143
Week5	255±35.35	60±36.05	0.041
Week6	275±35.35	55±35.35	0.015

## Discussion

Drinking sweetened AW as a non-alcoholic refreshment has been popular in various areas of Iran and the Middle East for ages owing to its pleasant flavor and therapeutic properties. The presence of EO within the AW is mostly responsible for its pleasant taste and odor. Table 4 summarizes the chemical compositions of ZM EOs from various studies. Carvacrol was determined to be the major ingredient of the EO isolated from AW in our investigation, which was comparable to the work done by Eftekhar et al. [ 23 ]. However, thymol was recognized as the second most common monoterpene in our study [ 24 ], despite being the most abundant phenolic monoterpene of ZM EO in the majority of previous reports with concentrations ranging from 13.1% to 66.9%. Differences in chemical composition between our study and others might be due to climatic change, geographical location, or developmental stage of the grown plant [ 25 ]. It has been shown that AW and EO samples were found to be rich in monoterpenes. However, the chemical composition of AW and EO could be different both qualitatively and quantitatively [ 26 ]. Inhibition of the pathogens’ growth by carvacrol has been reported in various articles [ 27 , 28 ]. Due to the hydrophobic nature of carvacrol (Log _Pmembrane/buffer_ =3.3), it has a stronger preference to accumulate in more hydrophobic regions such as the cytoplasmic membrane, causing an expansion of the membrane, inhibition of proton motive force, depletion of ATP pools, and finally cell death [ 29 - 31 ]. Thymol also demonstrated significant antimicrobial activity against different microorganisms [ 32 , 33 ]. Moreover, the possible synergistic effect of these monoterpenes also conformed with the ratio of 50%-50% of thymol- carvacrol [ 32 ]. Interestingly, it has been reported that the position of the hydroxyl group on the phenolic ring does not affect the antimicrobial activity. As a result, both thymol and carvacrol have hydroxyl groups at different positions of the phenolic ring, and the delocalized electron functional group was shown to be important for their effects on membrane properties, inhibition of ergosterol biosynthesis [ 34 ], alteration of membrane permeability, and leakage of ions and intracellular materials [ 35 ].

**Table 4:** Literature review on chemical comparison of the essential oil of *Zataria multiflora*

Compound	Compositions (%)			
	Sharififar *et al*. 41	Moazeni *et al*. 21	Eftekhar *et al*. 23	This study
Thymol	37.59	66.9	13.1	40.66
Carvacrol	33.65	15.2	50.57	46.55
γTerpinene	3.88	-	2.84	-
3Octanone	1.62	0.2	0.18	0.03
Linalool	1.75	0.4	1.27	1.33
αTerpineol	1.28	0.8	-	0.78
Carvone	-	7.35	7.3	4.44

The ZM EO has previously shown that significant antifungal and antibacterial activity *in vitro*. The AW demonstrated fungistatic and fungicidal effects against all *Candida* species tested in this investigation at doses ranging from 0.25 V/V to 0.5 V/V. The AW was rich in phenolic monoterpenes with recognized fungicidal properties, such as carvacrol and thymol [ 36 ]. As a result of the fungicidal properties of these AW components, the proximity of MIC and MFC values was predicted.

The moderate potency of AW in this study compared to EOs might be attributed to low levels of EO in AW compared to previous investigations. However, if the dilution factor (yield) of the EO in AW is considered, the inhibitory impact would be equal or perhaps superior to previous reports [ 11 ].

Moreover, in a previous study, it has been shown that the biofilm formation of *Candida* species was inhibited by the EO of *Mentha piperita* at concentrations up to 2 μl/ml [ 11 ]. In this study, AW successfully inhibited the formation of *Candida* biofilm at a concentration of 0.062 V/V (50%). Given the very low concentration of active compounds in the AW and its high potency, it can be concluded that the AW has a high potential to inhibit the formation of biofilm.

The stomach culture of infected mice indicated a significant decrease in gastric *Candida* colonization or even elimination (in one case) in those receiving AW of ZM. A decrease in the colonization rate was observed in the fourth week. The differences in mean CFU between mice treated with AW and untreated controls were statistically significant from the fifth week. Similarly, Khosravi et al treated disseminated candidiasis in BALB/c mice by ZM EO and found that *C. albicans* was cleared from the visceral organs in those treated by ZM EO. It should be noted that the active ingredient (i.e., EO) content of ZM AW was much lower than that used by Khosravi et al. [ 37 ]. This significant reduction in colonization rate by consumption of AW containing a trace amount of EO indicates the effectiveness of its ingredients such as thymol and cavacrol, which is consistent with other similar studies [ 38 , 39 ]. Our findings are consistent with histopathology investigations, which reveal that *Candida* colonization in mice decreases after administration of AW of ZM. To the best of our knowledge, this is the first report on the in vivo antifungal activity of ZM AW.

Furthermore, this herbal plant has been shown to significantly stimulate the immune system in experimental animals, suggesting that it could be used as an immunostimulatory agent [ 40 ].

## Conclusion

GC-MS analysis indicated that carvacrol and thymol, as oxygenated monoterpenes, were the most typical chemicals identified in AW of ZM in the current research. The presence of phenolic monoterpenes in the AW may be associated with the significant antifungal activity of AW, as determined by the elimination of *C. albicans* in the infected tissue. The antimicrobial and antioxidant properties of the AW led to the decrease of inflammation and the initiation of the healing process in the necrotic tissue. These findings can be added to the literature on the management of *C. albicans* infection to indicate that herbals can be a therapeutic choice in addition to the standard medicines.

## Ethical Considerations

This study conformed to the guidelines for the care and use of laboratory animals established by the Ethics Committee of Shiraz University (IR.SUMS.REC.1395.S521). Unnecessary animal suffering was avoided throughout the study.

## Acknowledgments

The authors would like to thank the support of the Research Deputy of Shiraz University of Medical Sciences (Grant No: 95-01-01-11403). The authors would also like to thank Omid Koohi-Hosseinabadi for his contribution to the animal experiments and Mohammad Bagher Dehghani for the preparation of histopathological slides. This study was derived from a thesis submitted in partial fulfillment for the degree of M.Sc. by Maryam Yazdanpanah. The abstract of this manuscript was presented at the 5th Iranian Congress of Medical Mycology.

## Authors’ contribution

M.Y. and collected A.I. and A.A.M. collected the aromatic water and distilled the EO. Z.Z.S. and A.I. analyzed EO data. A.A.M. performed the experiments. D.M. prepared and reported the histopathological data. K.Z., K.P., and M.Gh. participated in the study design, data analysis, and interpretation of results. All authors have read and approved this manuscript. 

## Conflicts of interest 

The authors declare that they have no competing interests.

## Financial disclosure

This study was part of the M.Sc. thesis of Maryam Yazdanpanah supported by a grant from Shiraz University of Medical Sciences, Shiraz, Iran (Grant No: 95-01-01-11403).
